# Met1-linked ubiquitination in cell signaling regulation

**DOI:** 10.52601/bpr.2024.230030

**Published:** 2024-08-31

**Authors:** Yanmin Guo, Yuqin Zhao, Yu-Sheng Cong

**Affiliations:** 1 Key Laboratory of Aging and Cancer Biology of Zhejiang Province, Hangzhou Normal University School of Basic Medical Sciences, Hangzhou 311121, China

**Keywords:** Ubiquitin, LUBAC, Met1-linked ubiquitination, Cell signaling

## Abstract

Met1-linked ubiquitination (Met1-Ub), also known as linear ubiquitination, is a newly identified atypical type of polyubiquitination that is assembled via the N-terminal methionine (Met1) rather than an internal lysine (Lys) residue of ubiquitin. The linear ubiquitin chain assembly complex (LUBAC) composed of HOIP, HOIL-1L and SHARPIN is the sole E3 ubiquitin ligase that specifically generates Met1-linked ubiquitin chains. The physiological role of LUBAC-mediated Met1-Ub has been first described as activating NF-κB signaling through the Met1-Ub modification of NEMO. However, accumulating evidence shows that Met1-Ub is broadly involved in other cellular pathways including MAPK, Wnt/β-Catenin, PI3K/AKT and interferon signaling, and participates in various cellular processes including angiogenesis, protein quality control and autophagy, suggesting that Met1-Ub harbors a potent signaling capacity. Here, we review the formation and cellular functions of Met1-linked ubiquitin chains, with an emphasis on the recent advances in the cellular mechanisms by which Met1-Ub controls signaling transduction.

## INTRODUCTION

Protein post-translational modifications (PTMs) refer to the covalent additions of functional groups or specific proteins to amino acids, which thereby extend the functional diversity and complexity of the proteome. To date, more than 450 types of PTMs have been identified (Venne *et al.* 2014). Among these, ubiquitination is one of the most widely studied PTMs, regulating nearly every aspect of cellular functions (Haglund and Dikic 2005). Among the eight types (Lys6, Lys11, Lys27, Lys29, Lys33, Lys48, Lys63 and Met1) of homogeneous polyubiquitin chains characterized, the Met1-Ub is the newly identified atypical type of polyubiquitination (Kirisako *et al.* 2006). The head-to-tail linear polyubiquitin chains have been originally considered as the translation product of *UBB* and *UBC* genes rather than the formation catalyzed by enzymes (Bianchi *et al.* 2015; Wiborg *et al.* 1985). Until 2006, Iwai and colleagues reported that LUBAC was a ubiquitin E3 ligase complex that possessed the feature to specifically assemble Met1-Ub (Kirisako *et al.* 2006). Later, Met1-Ub was demonstrated to activate the nuclear factor-κB (NF-κB) pathway (Tokunaga *et al.* 2009). Since then, Met1-Ub has attracted intense research attention to understand the mechanisms of its formation, regulation and biological functions. In this review, we describe the current insights into the conjugate and cleavage of Met1-linked ubiquitin chains with emphasis on the mechanisms of Met1-Ub in regulating cellular signal pathways.

## THE MET1-UB SYSTEM

Met1-Ub is linked via the regular peptide bond between the C-terminal carboxyl group of one ubiquitin and the α-NH2 group of Met1 of another ubiquitin. This process requires three enzymes, ubiquitin-activating enzyme (E1), ubiquitin conjugating enzyme (E2) and ubiquitin ligase (E3), among which E3s determine the specificity of ubiquitin linkage and substrate. To date, LUBAC is proven to be the only known E3 ubiquitin ligase capable of assembling Met1-Ub. OTU deubiquitinase with linear linkage specificity (OTULIN; also known as FAM105B or Gumby) and cylindromatosis (CYLD) are the DUBs to specifically disassemble these ubiquitin chains.

### Unique features of E3 ligase complex LUBAC

**Figure 1:**
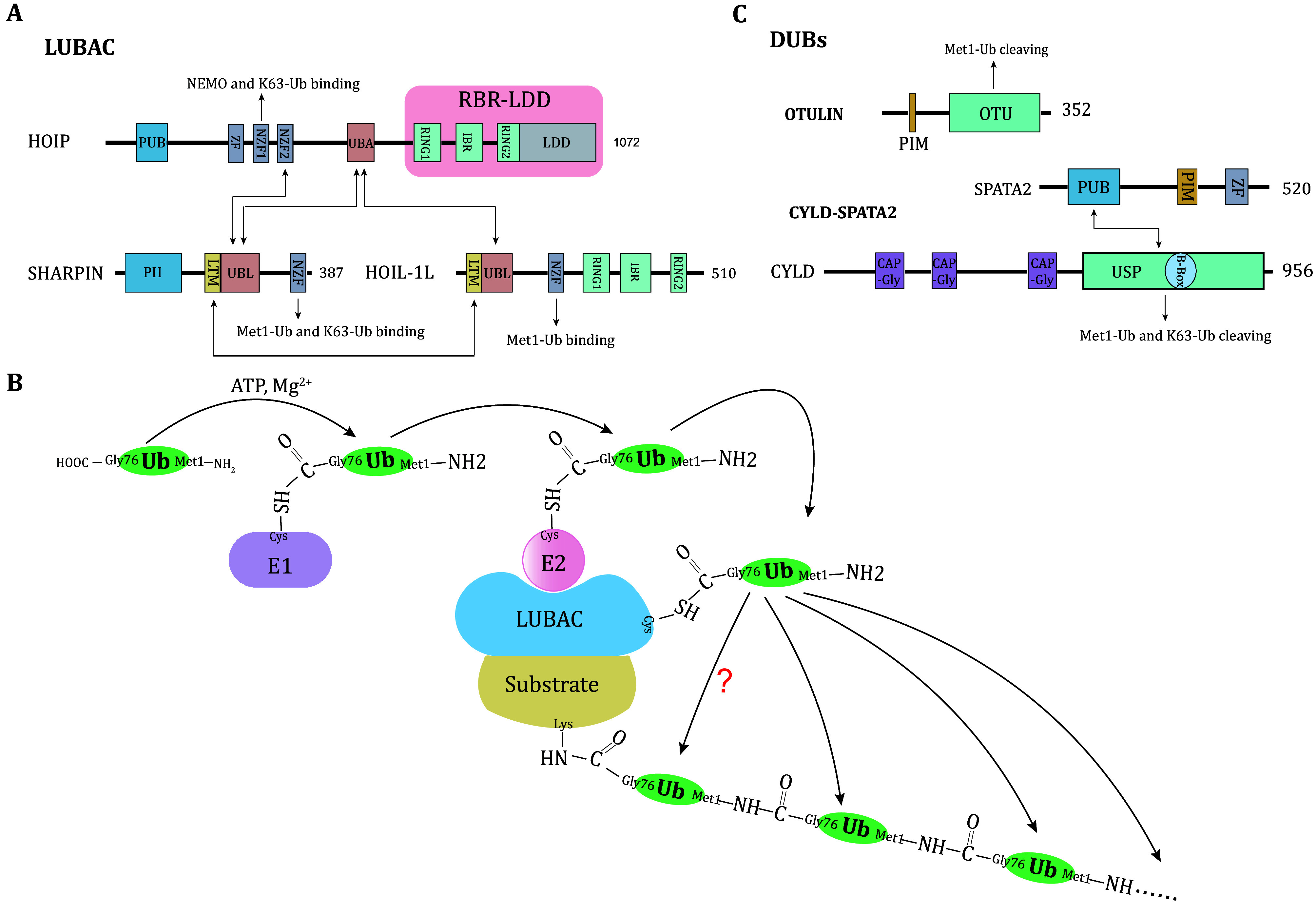
Met1-Ub system. **A** Schematic diagram of the LUBAC subunits, HOIP, HOIL-1L and SHARPIN. Arrows indicate interactions. **B** The model that LUBAC conjugates Met1-Ub to the substrate. E1 activates ubiquitin and leads to the formation of a thioester bond between the C terminus of ubiquitin and the catalytic cysteine of E1 (E1~Ub). Then the activated ubiquitin is transferred to the catalytic cysteine of E2 to form the E2~Ub thioester intermediate. Concomitantly, LUBAC binds E2~Ub and substrate to facilitate the transfer of ubiquitin molecules to the substrate. Arrows indicate the transfer of ubiquitin molecular. **C** Schematic diagram of LUBAC-associated DUBs, OTULIN and CYLD-SPATA2. Arrows indicate interactions. PUB, peptide:N-glycanase/ubiquitin-associated UBA-containing or UBX-containing protein; ZF, zinc finger; NZF, Npl4-like zinc finger; UBA, ubiquitin-associated; RING, really interesting new gene; IBR, in-between-RING; LDD, linear ubiquitin chain-determining domain; PH, pleckstrin homology; LTM, LUBAC-tethering motif; UBL, ubiquitin-like; PIM, PUB-interacting motif; OTU, ovarian tumour; CAP-Gly, cytoskeleton-associated protein glycine-rich; USP, Ubiquitin specific protease; B-box, B-box type zinc finger

In 2006, Kazuhiro Iwai’s team identified a novel E3 complex with a size of ~600 kDa and found that haem-oxidized IRP2 ubiquitin ligase 1L (HOIL-1L; also known as RBCK1) and HOIL1-interacting protein (HOIP; also known as RNF31) were the two crucial proteins for this complex formation. This E3 complex could specifically assemble N-terminal Met1-linked ubiquitin chains rather than Lys-linked types in conjunction with several E2s, such as E2-25K, UbcH5s and UbcH7. Considering the nature of this complex, they named it the linear ubiquitin chain assembly complex (LUBAC). In 2011, three research groups observed that SHANK-associated RH domain-interacting protein (SHARPIN; also known as SIPL1) was a novel subunit of LUBAC (Gerlach *et al.* 2011; Ikeda *et al.* 2011; Tokunaga *et al.* 2011). Thus, LUBAC is considered a ternary complex composed of HOIP, HOIL-1L and SHARPIN (Fig. 1A).

HOIP is the catalytically active subunit of LUBAC. The C-terminal RING1-IBR-RING2 (RBR) domain directly assembles Met1-Ub and is the enzymatically active catalytic domain. The linear ubiquitin chain-determining domain (LDD) is unique for HOIP and determines the specific formation of the Met1 linkage. However, when HOIP exists alone, it appears to exert little E3 activity due to the autoinhibited conformation by the intramolecular interaction between RBR and ubiquitin-associated (UBA) domains. The ubiquitin-like (UBL) domains of HOIL-1L and SHARPIN interact with the UBA domain of HOIP to release HOIP autoinhibition, contributing to full HOIP E3 ligase activity (Fujita *et al.* 2018; Tokunaga *et al.* 2009, 2011). In addition, LUBAC-tethering motifs (LTMs) in HOIL-1L and SHARPIN associate with each other to form a globular domain that is crucial in maintaining the stability of LUBAC (Fujita *et al.* 2018).

### The assembly of Met1-Ub

Like Lys-linked polyubiquitin chains, Met1-Ub also occurs through a sequential cascade of E1, E2 and E3. In the presence of Mg^2+^ and ATP, E1 activates a donor ubiquitin and transfers it to E2 to form E2~Ub thioester intermediate. Then RING1 domain of HOIP interacts with E2~Ub and transfers ubiquitin to the Cys885 site of its RING2 domain, forming an HOIP~Ub intermediate. Concomitantly, the C-terminal carboxyl group (-COOH) of donor ubiquitin is attached to the α-NH2 group of Met1 of acceptor ubiquitin, forming Met1-linked ubiquitin chains (Fig. 1B). The conserved RING2 has a role in positioning the acceptor ubiquitin and works with the LDD domain to lock the acceptor ubiquitin so that the α-NH2 of Met1 is the only one in proximity for the attack by donor ubiquitin rather than the ε-NH2 of any one of the seven lysine residues (Lechtenberg *et al.* 2016; Stieglitz *et al.* 2013).

Typically, ubiquitination occurs on the Lys residues of the substrate. Since HOIP targets the Met1 for ubiquitin conjugation, whether the Lys-linked ubiquitin on substrates is directly attached by HOIP remains controversial. It has been proposed that HOIP generates Met1-Ub using pre-existing Lys63-Ub as the acceptor, giving rise to hybrid Lys63/Met1-Ub chains (Emmerich *et al.* 2013, 2016). However, the E3(s) for the generation of pre-existing Lys63-Ub is not yet known. Recent studies have showed that HOIL-1L possesses the ability to conjugate monoubiquitin onto all LUBAC components, which is conducive to the formation of Met1-Ub mediated by HOIP (Fuseya *et al.* 2020), and HOIL-1L is responsible for the initiation of Met1-Ub by attaching first ubiquitin to the Lys residue of NEMO, a known substrate of LUBAC, and then HOIP is for the following Met1-Ub chain elongation (Smit *et al.* 2013). These suggest that HOIL-1L may play a key role in mediating the conjugation of ubiquitin onto the Lys residue of LUBAC’s substrates. Whether HOIL-1L or other E3 ligases are responsible for the initiation of Met1-Ub by attaching first ubiquitin to the Lys residue of substrates remains to be studied.

### The disassembly of Met1-Ub

The Met1-Ub system is tightly controlled by DUBs. Two DUBs have been reported to effectively hydrolyze Met1-Ub. They are OTULIN and CYLD (Fig. 1C). OTULIN belongs to the ovarian tumor (OTU) family of DUBs and is the only known DUB that exclusively disassembles Met1-Ub. Crystal structure reveals this specificity is due to a high affinity between the OTU domain of OTULIN and Met1 linkage, and a mechanism named “substrate-assisted catalysis” in which Glu16 residue on the proximal ubiquitin directly participates in the formation of the active site and activates OTULIN (Keusekotten *et al.* 2013). Besides the OUT catalytic domain, OTULIN contains a conserved PIM motif through which OTULIN is recruited to LUBAC by binding to HOIP PUB domain to antagonize LUBAC-mediated Met1-Ub (Schaeffer *et al.* 2014).

CYLD belongs to the ubiquitin specific protease (USP) family of DUBs and cleaves both Met1-Ub and Lys63-Ub. Since the structure of Met1-Ub is the most similar to that of Lys63-Ub among the eight types of ubiquitin chains, CYLD associates with and disassembles Met1-Ub and Lys63-Ub in a similar manner (Komander *et al.* 2009). In addition, CYLD also associates with HOIP. However, their interaction is indirect and requires the spermatogenesis-associated 2 (SPATA2) to act as the adaptor protein. The PIM motif and the PUB domain of SPATA2 binds to the PUB domain of HOIP and the USP domain of CYLD, respectively, which contributes to the formation of LUBAC-SPATA2-CYLD complex (Elliott *et al.* 2016).

## CELLULAR FUNCTIONS OF MET1-UB

**Figure 2:**
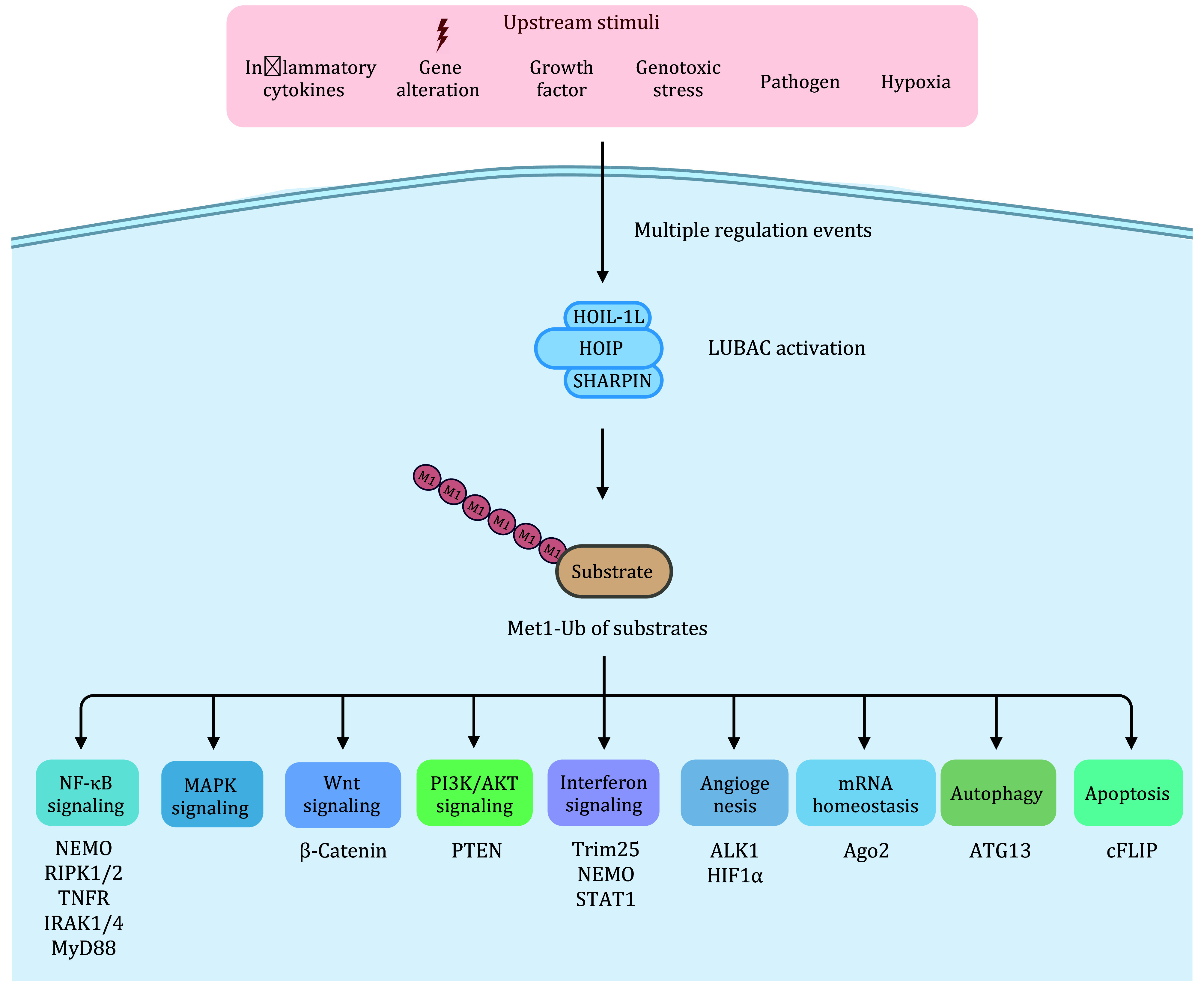
Functions of Met1-Ub in cell signaling. Various upstream stimuli lead to multiple regulation events that activate LUBAC. Then, LUBAC conjugates Met1-Ub on downstream substrates that are involved in the regulation of diverse cellular functions. The cell signaling regulated by Met1-Ub and a partial list of known substrates is shown at the bottom

Met1-Ub exerts its cellular roles largely by modifying substrates to affect protein activity, interaction, stability and subcellular localization (Table 1). So far, Met1-Ub has been demonstrated to be involved in some biological processes including NF-κB, mitogen-activated protein kinases (MAPK), Wnt/β-Catenin, the phosphatidylinositol 3-kinase/protein kinase B (PI3K/AKT), interferon (IFN) signaling, angiogenesis, mRNA homeostasis, protein quality control and so on (Fig. 2).

**Table 1:** The substrates of LUBAC

Substrate	Function of Met1-Ub	Reference
NEMO	Facilitating IKK activation	Tokunaga *et al*. (2009)
LUBAC	Destabilizing LUBAC components and impairing its ability	Keusekotten *et al*. (2013), Heger *et al*. (2018)
TRIM25	InducingTRIM25 degradation and inhibiting TRIM25 interaction with RIG-I.	Inn *et al*. (2011)
RIPK1	Promoting the recruitment of A20 to the TNF-RSC	Gerlach *et al*. (2011), Draber *et al*. (2015)
RIPK2	Promoting the recruitment of NEMO to facilitate IKK activation	Fiil *et al*. (2013)
TNFR	Promoting the recruitment of A20 to the TNF-RSC	Draber *et al*. (2015)
TRADD	Promoting the recruitment of A20 to the TNF-RSC	Draber *et al*. (2015)
IRAK1	Activating the canonical IKK complex	Emmerich *et al*. (2013)
IRAK4	Activating the canonical IKK complex	Emmerich *et al*. (2013)
MyD88	Activating the canonical IKK complex	Emmerich *et al*. (2013)
ASC	Regulating NLRP3 inflammasome activation	Rodgers *et al*. (2014)
FADD	Unknown (may affect cell survival and death.)	Goto and Tokunaga (2017)
Caspase-8	Unknown (may directly or indirectly inhibit caspase-8 activity)	Lafont *et al*. (2017)
ATG13	Stabilizing ATG13 protein and activating autophagy.	Chu *et al*. (2021)
cFLIP	Stabilizing cFLIP and protecting cells from TNFα-induced apoptosis	Tang *et al*. (2018)
PKC	Promoting degradation of activated PKC	Nakamura *et al*. (2006)
BCL10	Required for the association of BCL10 with NEMO and NF-κB activation	Yang *et al*. (2016)
STAT1	Inhibiting STAT1 binding to IFNAR2 and restricting STAT1 activation	Zuo *et al*. (2020)
STAT3	Inhibiting STAT3 activity by recruitment of the phosphatase TC-PTP to STAT3	Du *et al*. (2023)
Htt-polyQ	Promoting Htt-polyQ degradation and decreasing proteotoxicity	van Well *et al*. (2019)
Ago2	Restraining miRNA-mediated gene silencing	Zhang *et al*. (2021)
ALK1	Inhibiting ALK1 enzyme activity and Smad1/5 activation	Fu *et al*. (2021)
PTEN	Inhibiting PTEN phosphatase activity and promoting prostate cancer progression	Guo *et al*. (2022)
LKB1	Promoting LKB1 activity and AMPK activation	Chen *et al*. (2023)
GPX4	Stabilizing GPX4	Dong *et al*. (2022)
CENP-E	Facilitating the anchoring of CENP-E by KNL1 at attached kinetochores to promote chromosome congression and alignment	Wu *et al*. (2019)
HIF1α	Stabilizing HIF1α protein and promoting angiogenesis and lung tumorigenesis	Jin *et al*. (2024)

### NF-κB signaling

**Figure 3:**
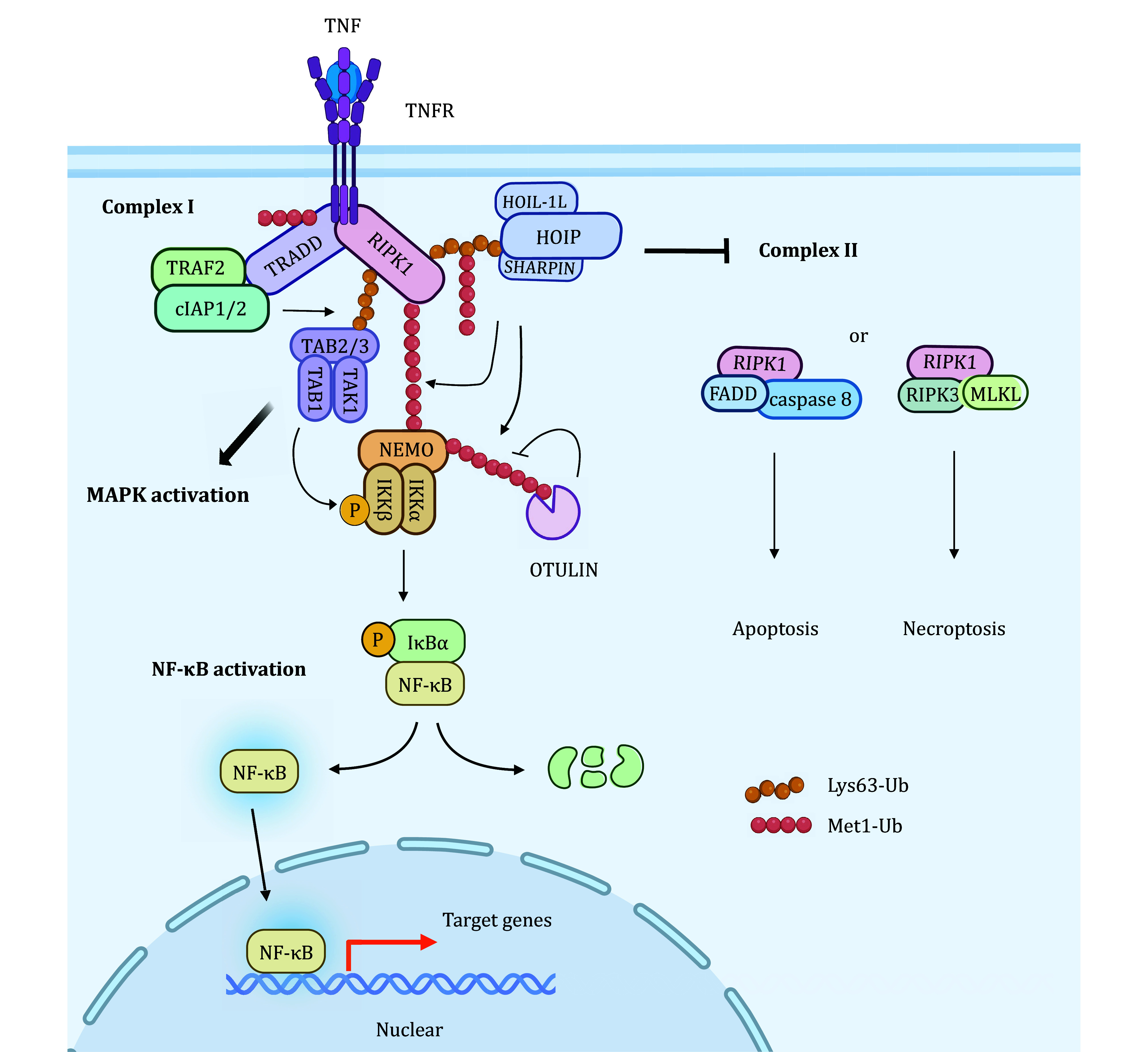
Met1-Ub plays a central role in TNF-induced NF-кB signaling and cell death. TNF stimulation triggers the recruitment of LUBAC to complex-I where it mediates the Met1-Ub of NEMO and other components of TNF-RSC. Met1-Ub of NEMO activates IKK and subsequent NF-кB signaling. Met1-Ub of the components of TNF-RSC prevents the formation of complex-II, thereby suppressing TNF-induced cell death, apoptosis and necroptosis. DUBs such as OTULIN and CYLD antagonize the actions of LUBAC by restricting the accumulation of Met1-Ub. LUBAC recruitment also ensures the full activation of the MAPK pathway. Different ubiquitin linkage types are indicated in the figure

Met1-Ub has been extensively studied in activating NF-κB signaling in response to tumor necrosis factor (TNF). Upon TNFα stimulation, TNF receptor (TNFR) recruits TNFRSF1A associated via death domain (TRADD), TNF receptor associated factor 2 (TRAF2), receptor-interacting protein kinase 1 (RIPK1) and cellular inhibitor of apoptosis protein 1/2 (cIAP1/2) to form TNF receptor signaling complex (TNF-RSC; named complex-I). Within this complex, the E3 ligase cIAP1/2 conjugates the Lys63-Ub on RIPK1 or other substrates. Subsequently, LUBAC is recruited to TNF-RSC by recognizing the Lys63-Ub through the NZF domains of HOIP and SHARPIN, and conjugates Met1-Ub on RIPK1, TRADD, TNFR and itself. The Lys63-Ub and Met1-Ub assembled by cIAP1/2 and LUBAC serve as the adaptors for the next recruitment of two kinase complexes, the transforming growth factor-β (TGF-β)-activated kinase 1/TAK1-binding proteins (TAK1/TAB) and IKK, respectively. The recruitment of IKK results in the Met1-Ub of NEMO by LUBAC and phosphorylation of IKKβ by TAK1/TAB, which promotes IKK activation (Zhang *et al.* 2014). In turn, IKK phosphorylates IκBα, leading to its degradation by the proteasome. Thus, free NF-κB are released from IκBα and translocate into the nucleus where they drive transcription of multiple responsive genes (Fig. 3). Once any one of the LUBAC components is absent, the Met1-Ub level of RIPK1 will decrease, which may cause the dissociation of RIPK1 from complex-I, leading to the destabilization of complex-I and the formation of complex-II (Gerlach *et al.* 2011; Haas *et al.* 2009; Ikeda *et al.* 2011; Tokunaga *et al.* 2011). In response to TNF, complex-II can induce cell death through apoptosis or necroptosis (Fig. 3). Therefore, LUBAC-mediated Met1-Ub is crucial for maintaining NF-κB signaling through activating IKK and stabilizing complex-I.

LUBAC-associated DUBs can negatively regulate NF-κB signaling by inhibiting Met1-Ub (Verboom *et al.* 2021). OTULIN removes the Met1-Ub from TFNR-RSC, such as RIPK1 and NEMO, counteracting Met1-Ub-mediated NF-κB signaling (Fiil *et al.* 2013; Keusekotten *et al.* 2013). CYLD can remove both Lys63-Ub and Met1-Ub from RIPK1 and NEMO, and thereby suppressing the activation of the NF-κB (Draber *et al.* 2015). A20 (also known as TNFAIP3) suppresses Met1-Ub-mediated NF-κB activation by disrupting the binding of NEMO to LUBAC via interacting with the Met1-Ub utilizing its ZF7 domain (Tokunaga *et al.* 2012). Furthermore, PTMs can fine-tune the functions of HOIP to control LUBAC-mediated NF-κB signaling. Phosphorylation of HOIP at Ser1066 in the LDD region by MST1 attenuates LUBAC E3 ligase activity and negatively regulates the NF-κB-dependent inflammatory gene expression induced by TNFα (Lee *et al.* 2019). Ubiquitination of the HOIP Lys1056 site causes a conformational change of HOIP to block LUBAC activity and ultimately terminate LUBAC-mediated NF-κB signaling (Bowman *et al.* 2015).

In addition to TNFR, LUBAC-mediated Met1-Ub is also involved in NF-κB signaling triggered by interleukin 1 receptors (IL-1Rs) (Tokunaga *et al.* 2009), nucleotide-binding oligomerisation domain 2 (NOD2) (Damgaard *et al.* 2012), Toll-like receptors (TLRs) (Zinngrebe *et al.* 2016), cluster of differentiation 40 (CD40) (Ikeda *et al.* 2011), T cell receptors (TCRs) (Yang *et al.* 2016), epidermal growth factor receptor (EGFR) (Hua *et al.* 2021) and DNA damage (Niu *et al.* 2011). Although the stimuli and receptors vary, signaling cascades share a similar architecture as follows: (1) the formation of receptor signaling complexes (RSCs); (2) the recruitment of LUBAC to RSCs; (3) LUBAC-mediated Met1-Ub of NEMO and other complements of RSCs; (4) IKK and subsequent NF-κB activation.

### MAPK signaling

In response to TNF stimuli, LUBAC enhances the stabilization of TNF-RSC and subsequent TAK1 activation that mediates not only the NF-κB signaling but also the MAPK cascades. Depletion of any of the LUBAC components has been shown to reduce the activation of MAPK signaling in response to inflammatory cytokines in different cell lines, indicating that Met1-Ub is necessary for the activation of MAPK signaling (Chen *et al.* 2019; Haas *et al.* 2009). However, some studies have reported that the activation of the c-Jun aminoterminal kinase (JNK) and ERK is unchanged, even slightly enhanced, in the absence of either HOIL-1L or SHARPIN (Tokunaga *et al.* 2009, 2011). Importantly, MEF cells from knockin mice expressing HOIP^C879S^ (the inactive mutant) show unchanged activation of JNK and p38 MAPK signaling in response to IL-1α (Zhang *et al.* 2014), appearing that HOIP E3 activity is dispensable for MAPK activation. Thus, the roles of Met1-Ub in MAPK signaling are still controversial and the underlying mechanism by which Met1-Ub regulates MAPK signaling requires further studies.

### Wnt/β-Catenin signaling

Studies have shown that Wnt/β-Catenin signaling may be negatively regulated by LUBAC and positively regulated by OTULIN, emphasizing that the balance of the Met1-Ub system is critical for maintaining Wnt/β-Catenin signaling. HOIP knockdown in adrenocortical carcinoma cells is shown to affect the Wnt pathway target gene expression (Ehrlund *et al.* 2012). Notably, homozygous mutant mice carrying the loss-of-function mutations in *OTULIN* (W96R and D336E) are embryonically lethal due to the defective Wnt/β-Catenin signaling and angiogenesis, characterized by the accumulation of Met1-Ub (Rivkin *et al.* 2013). OTULIN interacts with disheveled 2 (DVL2) to promote Wnt signaling and binds to the HOIP to antagonize LUBAC-mediated inhibition of Wnt signaling (Rivkin *et al.* 2013; Takiuchi *et al.* 2014). Moreover, OTULIN phosphorylation at Tyr56 by the tyrosine protein kinase ABL1 enhances its interaction with β-catenin while diminishing its association with HOIP, which inhibits β-catenin Met1-Ub and its degradation to promote Wnt/β-Catenin activation (Wang *et al.* 2020).

### PI3K/AKT signaling

The indication that Met1-Ub is involved in PI3K/AKT signaling comes from the observations that HOIP and SHARPIN interact with phosphatase and tensin homolog deleted on chromosome 10 (PTEN) to reduce PTEN function to potentiate PI3K/AKT signaling (De Melo *et al.* 2014a, b; Niu *et al.* 2021). The direct evidence for Met1-Ub regulating PI3K/AKT signaling is derived from the identification that PTEN is directly modified by Met1-Ub (Guo *et al.* 2022). Mechanistically, LUBAC (HOIP+SHARPIN) conjugates Met1-Ub chains to PTEN, which significantly inhibits PTEN phosphatase activity and accelerates the activation of PI3K/AKT signaling, promoting prostate cancer progression (Guo *et al.* 2022). Thus, LUBAC-mediated Met1-Ub is a critical signal mediator in PI3K/AKT activation and tumor progression.

### Interferon (IFN) signaling

Met1-Ub shows an important role in antiviral responses through suppressing IFN signaling. One study found that Met1-Ub chains were conjugated on the tripartite motif-containing protein 25 (TRIM25) to induce TRIM25 degradation and inhibit its interaction with retinoic acid-inducible gene I (RIG-1), thereby negatively regulating RIG-I-mediated type I IFN induction (Inn *et al.* 2011). Another study suggested that Met1-Ub of NEMO competed with the mitochondrial antiviral signaling protein (MAVS) to bind TNF receptor-associated factor 3 (TRAF3), disrupting the interferon regulatory factor 3 (IRF3) signaling cascade and reducing IFN production (Belgnaoui *et al.* 2012). Moreover, Met1-Ub was reported to restrict the signal transducer and activator of transcription 1 (STAT1) activation, thereby inhibiting antiviral IFN signaling (Zuo *et al.* 2020).

### The emerging functions of Met1-Ub

Recent work has revealed that Met1-Ub also has other cellular functions besides the above mentioned. The role of Met1-Ub in regulating angiogenesis is illustrated in mouse models, in which mice harboring OTULIN mutant or deficiency are embryonic lethal due to angiogenesis defects (Fu *et al.* 2021; Rivkin *et al.* 2013). The possible mechanism is that LUBAC mediated the Met1-Ub of the activin receptor-like kinase 1 (ALK1) to inhibit ALK1 enzyme activity and Smad1/5 activation, leading to the abnormal expression of downstream target genes that mediate vasculature and angiogenesis (Fu *et al.* 2021). Conversely, OTULIN deubiquitinates ALK1 to promote its activation, thus governing angiogenesis (Fu *et al.* 2021). Notably, studies reveal that Met1-Ub can act as a degradation signal to control protein quality (van Well *et al.* 2019), although previous studies suggest that Met1-Ub more inclines to be a cellular transduction signal. LUBAC promotes TRIM25 Met1-Ub and induces its proteasomal degradation, suppressing IFN-mediated antiviral signaling (Inn *et al.* 2011). Further, HOIP is reported to be recruited to the misfolded Huntingtin with an expanded polyglutamine tract (Htt-polyQ) in a p97/VCP-dependent manner, promoting Met1-Ub of Htt-Q97 and subsequent degradation, and decreasing proteotoxicity (van Well *et al.* 2019). And LUBAC itself also undergoes Met1-Ub-mediated proteasomal degradation. Increased auto-Met1-Ub and subsequent degradation of LUBAC have been reported in some OTULIN-deficient cells (Damgaard *et al.* 2019; Heger *et al.* 2018). In addition, Met1-Ub is also involved in autophagy. Study shows that Met1-Ub promotes autophagy initiation and maturation by controlling the Met1-Ub of autophagy-related protein 13 (ATG13) (Chu *et al.* 2021). Recently, Met1-Ub has been demonstrated to control chromosome alignment during mitosis (Wu *et al.* 2019). LUBAC targets the kinetochore motor CENP-E for Met1-Ub, which promotes the recruitment of CENP-E by KNL1, a newly identified receptor for Met1-Ub, to facilitate chromosome congression and dynamic chromosome alignment. Although Met1-Ub has been reported to be involved in diverse cellular processes (Fig. 2), the physiological functions and precise mechanisms merit a future investigation.

## SUMMARY AND PERSPECTIVES

During the past dozen years, many advances have been made in understanding of the principle for formation and disassembly of Met1-Ub. The LUBAC/DUBs act as the on/off switch in controlling the Met1-Ub system, thereby regulating protein function and allowing for a rapid response to stimuli. Undoubtedly, strict regulation of LUBAC and its associated DUBs is critical for normal Met1-Ub functions. However, there are few researches to study how the activity and function of LUBAC are regulated. In addition, it is evident that the recruitment of LUBAC is conducted differently depending on variable stimuli, how it is specifically recruited to cellular locations requires further investigation.

Met1-Ub was originally identified to activate NF-κB signaling to regulate inflammation and immunity responses and it has since been found to regulate other signaling pathways and physiologic processes (see the previous section). Given that Met1-Ub exerts diverse biological functions mainly through modifying target proteins, exploring new substrates for Met1-Ub is necessary to better understand its cellular function and mechanism. In mice, a deficiency of *HOIP* causes embryonic lethality (Peltzer *et al.* 2014), and *SHARPIN* deficient mice exhibit severe chronic proliferative dermatitis (Seymour *et al.* 2007). In human, aberrance of Met1-Ub cascade is implicated in a variety of diseases, including cancer (Jimbo *et al.* 2023; Yang *et al.* 2014), autoinflammation (Boisson *et al.* 2015), immunodeficiency (Boisson *et al.* 2015; Oda *et al.* 2019), amylopectinosis (Boisson *et al.* 2012), polyglucosan storage myopathy (Nilsson *et al.* 2013) and Alzheimer's disease (Asanomi *et al.* 2022). These results imply that the LUBAC activity is required for the maintenance of normal physiology. Many LUBAC inhibitors have been identified and reported to effectively suppress Met1-Ub and regulate cell signaling (Fujita *et al.* 2018; Hua *et al.* 2021; Katsuya *et al.* 2019), suggesting new strategies for diagnosis and therapeutic intervention in diseases by targeting Met1-Ub.

## Conflict of interest

Yanmin Guo, Yuqin Zhao and Yu-Sheng Cong declare that they have no conflict of interest.
